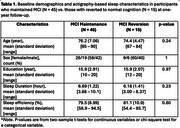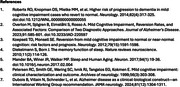# Sleep's role in MCI reversion: A One‐Night Impact

**DOI:** 10.1002/alz70857_098232

**Published:** 2025-12-24

**Authors:** Jun Ha Chang, Melissa Hatch, Karla Lynch, Vaishali S Phatak, Daniel L Murman, Matthew Rizzo

**Affiliations:** ^1^ University of Nebraska Medical Center, Omaha, NE, USA

## Abstract

**Background:**

Mild cognitive impairment (MCI) can revert to normal cognitive function in 15‐48 % of cases^1‐3^, suggesting that some initial MCI diagnosis reflect transient or context‐dependent conditions. Sleep disruption is common in older adults, and even one night of poor sleep can adversely affect cognitive performance temporarily.^4,5^ We hypothesized that the greater sleep disruption on the night preceding cognitive assessments would be associated with increased likelihood of MCI reversion at one‐year follow‐up.

**Method:**

Sixty older adults (mean age = 75 years; 25 males), meeting Petersen's criteria^6^ for MCI based on a battery of five cognitive‐domain assessments (memory, attention, visuospatial, language, and executive function) at baseline year, participated in the study. Participants wore wrist‐ actigraphs to collect objective sleep data and completed sleep diaries to validate time to go to bed and out of bed on the night before the assessments. Sleep duration (hours) and efficiency (%) were standardized across individuals using Z‐scores. A logistic regression model, adjusted for age and gender, estimated the odds of maintaining MCI classification versus reverting to normal cognitive status at a one‐year follow‐up using the same battery.

**Result:**

At one‐year follow‐up, 45 (75%) maintained MCI status and 15 participants (25%) reverted to normal status (See details in Table 1). Shorter sleep duration the night before the baseline cognitive assessments was significantly associated with higher odds of reversion (b = ‐0.76, *p* =  0.049). Each standard deviation (1.27 hour) increases in sleep duration lowered the odds of reversion by approximately 53%. Sleep efficiency, age, and gender were not significant predictors (*p* > 0.10).

**Conclusion:**

In this preliminary study, participants who slept shorter than the group average on the night before cognitive examination were more likely to revert from MCI to normal cognitive classification one year later. These findings underscore the need to consider acute sleep variation in MCI diagnoses. Incorporating short‐term sleep measures into clinical care and trials^7^ along with biological biomarkers may help refine diagnostic accuracy as prodromal Alzheimer's vs. potentially “reversible” causes, and guide targeted interventions for cognitive health.